# Biosynthetic Gene Cluster Diversity and Species-Specific Metabolic Potential in Ustilaginaceae

**DOI:** 10.3390/jof12050319

**Published:** 2026-04-27

**Authors:** Chao Lin, Zhenxin Wang, Na Zhang, Yuying Liu, Lixiao Song, Jin Zhang, Khassanov Vadim, Haiqiang Wang, Minglei Li, Jianzhao Qi

**Affiliations:** 1 Shaanxi Key Laboratory of Natural Products & Chemical Biology, College of Chemistry & Pharmacy, Northwest A&F University, Yangling, Xianyang 712100, China; 2 Center of Edible Fungi, Northwest A&F University, Yangling, Xianyang 712100, China; 3 School of Soil and Water Conservation Science and Engineering, Northwest A&F University, Yangling, Xianyang 712100, China; 4 Department of Plant Protection and Quarantine, Faculty of Agronomy, S. Seifullin Kazakh Agrotechnical Research University, Zhenis Avenue, Astana 010011, Kazakhstan

**Keywords:** Ustilaginaceae, biosynthetic gene clusters, secondary metabolites, plant pathogen

## Abstract

Plant pathogens pose a severe threat to global agricultural production, and their pathogenicity is closely linked to the biosynthesis of secondary metabolites. Basidiomycete within the family Ustilaginaceae represent significant plant pathogens, among which *Ustilago maydis*, as a model species, has been extensively studied for its secondary metabolites. However, the biosynthetic potential of other species within this family remains poorly understood. In this study, we conducted whole-genome bioinformatic analyses of 16 Ustilaginaceae species, including *U. maydis*, to systematically identify the distribution of biosynthetic gene clusters (BGCs), core gene domain compositions, and interspecies similarities. A total of 181 predicted BGCs were identified, averaging approximately 11 per species. BGCs for mannosylerythritol lipids (MELs), siderophores, and itaconic acid, as well as the melanin-associated genes *pks*1 and *pks*2, were widely distributed across most species. Conversely, an additional melanin biosynthetic gene cluster was found exclusively in *U. maydis* strain 521, indicating species-specific occurrence. Furthermore, this study identified a novel class of polyketide synthase (PKS) gene clusters with uncharacterized functions across 15 species, exhibiting high sequence and structural conservation between species. These findings reveal the rich metabolic diversity and species-specific biosynthetic potential of Ustilaginaceae, and by using *U. maydis* as a reference model, we highlight several BGCs (e.g., for MELs, siderophores, itaconic acid, and melanin) that are known to contribute to virulence or pathogenicity in plant hosts. This provides new insights into their pathogenic mechanisms.

## 1. Introduction

Plant diseases, particularly those caused by pathogenic microorganisms, pose a significant threat to global agricultural production [1,2]. With the intensification and globalization of modern agriculture, the transmission rate and geographic range of plant pathogens have expanded considerably, resulting in increasingly severe crop losses. These pathogens not only impair normal plant growth and reduce yields but may also compromise the quality of agricultural products, thereby threatening food security and destabilizing market supply [3]. Pathogen infection typically induces growth retardation, wilting, or mortality in host plants. Root rot and leaf spot represent among the most prevalent diseases caused by fungal and bacterial pathogens, affecting not only individual crop productivity but also potentially disrupting species balance within agroecosystems [4,5]. Fungi constitute one of the primary classes of plant pathogens [6,7], notorious for their capacity to damage or eliminate plant populations in natural and cultivated ecosystems [8]. These pathogenic fungi deploy diverse infection strategies, with plant-fungal interactions spanning a continuum from beneficial symbiosis to host mortality [6,9]. Among the numerous phytopathogenic fungi, members of the family Ustilaginaceae represent a particularly significant group, capable of infecting major cereal crops, including maize, sorghum, and wheat, thereby causing substantial economic losses.

As the most extensively studied model species within the family Ustilaginaceae, *U. maydis* is a devastating pathogen capable of infecting multiple organs of maize plants, including leaves, stems, and inflorescences [10,11,12]. *U. maydis* can infect maize at all developmental stages, inducing tumorous growths, chlorosis, and tissue necrosis, thereby substantially compromising both yield and grain quality [13]. This pathogen exhibits rapid dissemination and high virulence, posing a significant threat to maize production worldwide. Outbreaks of *U. maydis* not only diminish the economic value of affected crops but may also cause broader ecological impacts, thereby undermining the sustainability of agricultural systems. As a well-established model for phytopathogenic fungi, *U. maydis* possesses a set of BGCs that produce secondary metabolites directly linked to plant infection, including MELs involved in immune suppression, siderophores for iron acquisition during colonization, itaconic acid modulating host redox balance, and melanin for stress tolerance and virulence. These BGCs serve as a reference framework to compare related species within Ustilaginaceae.

Secondary metabolites, including phytotoxins and effector compounds, can perturb plant immune responses, impair cellular functions, and disrupt physiological homeostasis, thereby increasing host susceptibility to infection and inducing pathological symptoms [14]. During its life cycle, *U. maydis* synthesizes diverse secondary metabolites that modulate host physiological processes, thereby facilitating the establishment and proliferation of parasitic colonization. Although the secondary metabolite profile of *U. maydis* has been partially characterized, the biosynthetic potential of the majority of Ustilaginaceae species remains largely unexplored. Therefore, this study employed comparative genomic and bioinformatic approaches to analyze 16 species within the family Ustilaginaceae, using *U. maydis* as a reference to specifically examine the distribution of BGCs that are known or predicted to contribute to pathogenicity (e.g., MELs, siderophores, itaconic acid, melanin). We systematically investigated the distribution of these BGCs across species and characterized the domain architecture of core biosynthetic enzymes. Genes associated with melanin biosynthesis (*pks*1 and *pks*2), together with BGCs for MELs, siderophores, and itaconic acid, were widely distributed among most species analyzed. Conversely, a separate gene cluster dedicated to melanin synthesis (distinct from the widely distributed *pks*1/*pks*2) was exclusively identified in *U. maydis* strain 521, demonstrating pronounced species specificity. Furthermore, we discovered a conserved class of previously uncharacterized PKS gene clusters across 15 species, exhibiting high sequence similarity and syntenic organization. These findings provide novel insights into the secondary metabolic repertoire of Ustilaginaceae fungi and establish a foundation for the heterologous expression and metabolic engineering of bioactive compounds.

## 2. Materials and Methods

### 2.1. Strains and Genomic Sequences

The 16 species analyzed in this study represent all Ustilaginaceae species for which complete or draft genome assemblies were publicly available in the NCBI database at the time of analysis. This dataset includes both well-characterized plant pathogens (e.g., *U. maydis*, *U. hordei*) and non-pathogenic, reflecting the current scope of genomic resources for this family (Table S1).

### 2.2. Gene Cluster Prediction and Similarity Network Analysis

BGCs were predicted across the 16 Ustilaginaceae genomes using antiSMASH 7.0 [15]. Input files consisted of genome sequences in FASTA format together with corresponding gene annotation files in GFF3 format. The analysis was performed using the following parameters: relaxed detection stringency with all additional and time-consuming features enabled. Predicted BGCs were classified into three major categories: nonribosomal peptide synthetases (NRPS, including NRPS-like), PKS, and terpene synthases. A BGC similarity network was constructed across all genomes using BiG-SCAPE v1.1.5 with the following command: bigscape. py -i input -o output -cutoffs 0. 5 -mibig21. In this network, each node represents an individual BGC, and edges connect nodes sharing similar Pfam domain architectures [16]. A similarity cutoff of 0.5 was applied, and the resulting network was visualized using Cytoscape v3.9.1 (https://cytoscape.org, accessed on 27 May 2024).

### 2.3. Sequence Identity Analysis and Structural Analysis of Multi-Domain Enzymes

To analyze sequence similarity among core biosynthetic enzymes, protein sequences were aligned using Clustal Omega v1.2.4 (https://www.ebi.ac.uk/Tools/msa/clustalo/, accessed on 5 July 2024), and pairwise percentage identity matrices were generated. For detailed characterization of PKS, NRPS, and NRPS-like enzymes, domain architecture was analyzed using the Synthaser web server [17]. The examined domain types included: adenylation (A), acyl carrier protein (ACP), acyltransferase (AT), thiolation (T), condensation (C), ketosynthase (KS), ketoreductase (KR), dehydratase (DH), enoylreductase (ER), thioesterase (TE), product template (PT), starter unit: acyl carrier protein transacylase (SAT), 4′-phosphopantetheinyl transferase(MPT) and phosphopantetheinyl transferase (PPT).

### 2.4. Homology and Similarity Analysis of BGCs

To compare synteny and sequence similarity among BGCs predicted by antiSMASH, homology relationships were assessed using Clinker v0.0.32 [18]. This tool evaluates pairwise similarity between gene clusters based on encoded protein sequence alignments, with the integrated clustermap.js module employed for visualization of gene cluster comparison maps.

## 3. Results

### 3.1. Prediction and Classification of BGCs in Ustilaginaceae

**Figure 1:**
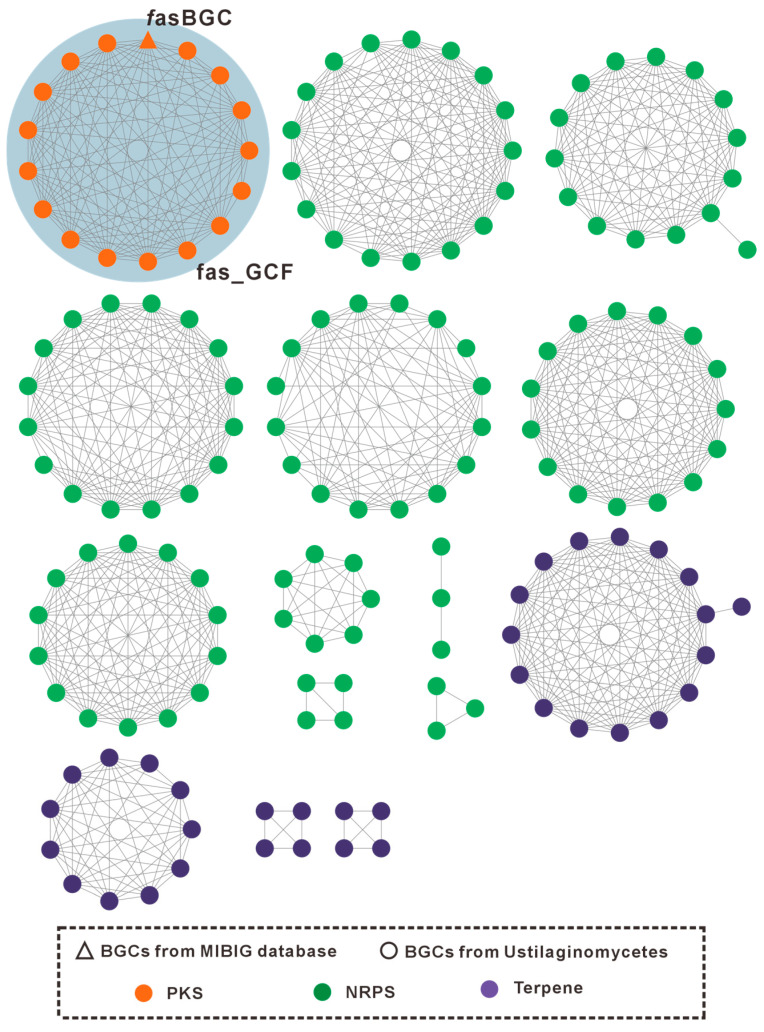
GCF network of Ustilaginaceae BGCs. Triangles and circles represent reference BGCs from the MIBiG database and BGCs identified from the 16 analyzed Ustilaginaceae genomes, respectively. Distinct colors indicate different BGC classes: green for NRPS, purple for terpene synthases, and orange for PKS.

To comprehensively characterize BGCs within the family Ustilaginaceae, antiSMASH 7.0 was employed to predict BGCs across 16 genomes. A total of 181 predicted BGCs were identified (Table S1), distributed relatively evenly among species, with an average of approximately 11–12 BGCs per genome (Figure 1). These findings indicate that Ustilaginaceae species possess substantial biosynthetic potential.

To further elucidate the characteristics of these BGCs, gene cluster family (GCF) network analysis was performed using BiG-SCAPE. Based on the similarity of Pfam domain compositions within predicted biosynthetic enzymes, BiG-SCAPE classified 181 BGCs derived from the 16 Ustilaginaceae genomes and 1035 reference BGCs from MIBiG 2.1 into 15 GCFs and 1053 singleton clusters (Figure 1, isolated nodes omitted; Figure S1). Within the GCF network, BGCs of distinct chemical classes formed separate subnetworks, including 1 GCF consisting entirely of type I PKS clusters, 10 GCFs comprising exclusively NRPS clusters, and 4 GCFs containing only terpene clusters.

Using antiSMASH and BiG-SCAPE, we first catalogued the overall BGCs composition across the studied genomes. With *U. maydis* serving as a reference, we then proceeded to examine specific secondary metabolite pathways with established or proposed roles in plant infection, regardless of their BGCs classification. This includes pathways associated with siderophores (NRPS-derived), melanin (PKS-derived), MELs, cellobiose lipids, and itaconic acid. The detailed characterization of these pathways is presented in the following sections.

### 3.2. Cellobiose-Lipids and Their Biosynthetic Pathways

Ustilagic acids are rare glycolipid biosurfactants composed of a cellobiose disaccharide core glycosidically linked to long-chain hydroxy fatty acids, specifically 15,16-dihydroxypalmitic acid (ustilagic acid A) or 2,15,16-trihydroxypalmitic acid (ustilagic acid B) [19,20,21]. The disaccharide moiety is further acylated with short-chain fatty acids (typically acetate or butyrate) at the 2′- and 6′-positions. Additionally, a novel analogue, ustilagic acid C, bearing distinct acylation patterns, has been characterized in *U. maydis* [22]. Ustilagic acid represents the first microbial glycolipid discovered with fibroside-like structural features and is regarded as a signature metabolite of the species *U. maydis* and the genus *Ustilago*.

**Figure 2:**
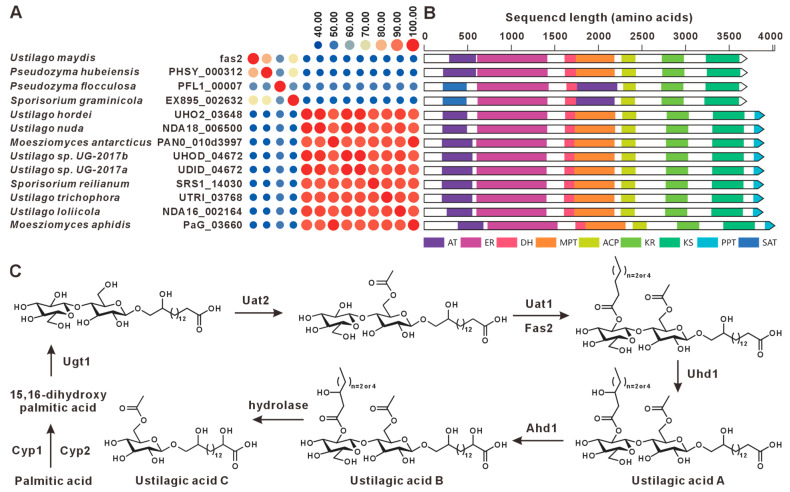
Ustilagic acid compound biosynthesis. (**A**) Comparison of the amino acid sequence identity of fas2 and its homologues. (**B**) Domain comparison of fas2 and its homologues. The following abbreviations for protein domains are predicted by synthaser: AT, acyltransferase; ER, enoylreductase; DH, dehydratase; MPT, 4′-phosphopantetheinyl transferase; ACP, acyl carrier protein; KR, ketoreductase; KS, ketosynthase; PPT, phosphopantetheinyl transferase; SAT, starter unit acyltransferase. (**C**) The biosynthetic pathway for ustilagic acid.

**Figure 3:**
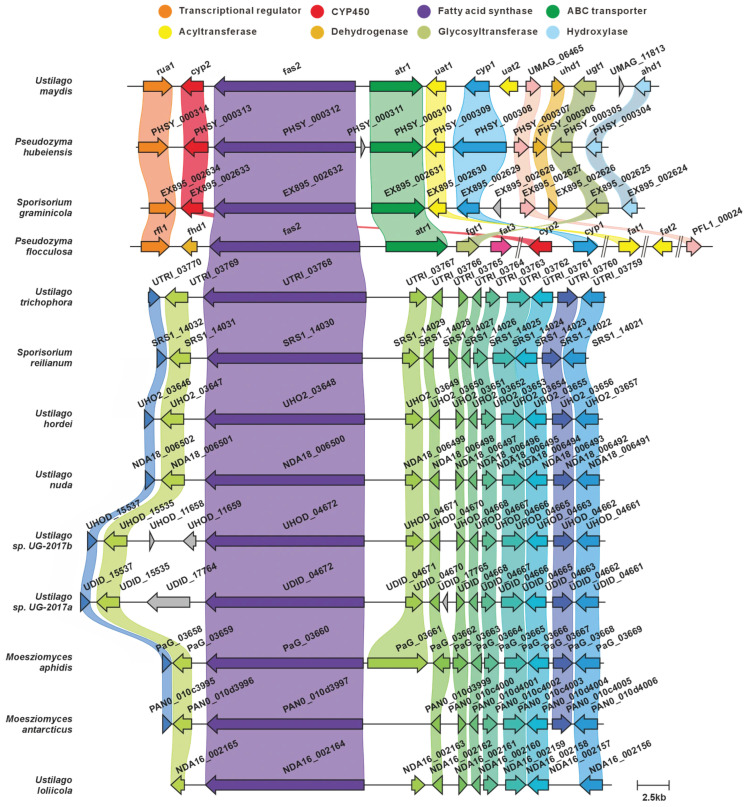
Comparison of the BGC for ustilagic acid and its similar BGCs: homologous genes are connected by a band of the same color.

The BGC governing ustilagic acid production in *U. maydis* comprises nine genes, including one transcriptional regulator (rua1) and nine structural genes (*cyp*2, *fas*2, *atr*1, *uat*1, *cyp*1, *uat*2, *uhd*1, *ugt*1, *ahd*1) [23,24]. This study identified twelve *fas2* paralogs across the analyzed genomes, with all fatty acid synthase (fas2) gene products exhibiting >44% pairwise sequence identity (Figure 2A, Table S2). Domain architecture analysis revealed highly conserved domain compositions across these sequences (Figure 2B). Comparative BGC analysis indicated that only the clusters from *U. maydis*, *Pseudozyma hubeiensis*, *Sporisorium graminicola*, and *Pseudozyma flocculosa* exhibited high synteny and sequence similarity. Unfortunately, we did not identify homologs of the nine structural genes in the vicinity of the *fas*2 orthologs in any other species examined. Notably, *U. maydis* and *P. flocculosa* are responsible for the biosynthesis of ustilagic acid and flocculosin, respectively (Figure 3) [24]. We therefore infer that these four species possess the genetic capacity for ustilagic acid and related glycolipid biosynthesis, whereas the BGCs in the remaining species likely participate in the biosynthesis of alternative fatty acid-derived metabolites. The biosynthetic pathway for ustilagic acid has been previously elucidated, illustrating the functional roles of cluster-encoded genes (Figure 2C) [23].

### 3.3. MELs and Their Biosynthetic Pathway

MELs are well-characterized biosurfactants originally discovered in 1955 from the fermentation broth of *U. maydis* and related Ustilaginaceae, exhibiting significant biotechnological potential [25,26]. The molecular scaffold consists of a disaccharide backbone comprising mannose and erythritol, with the C2 and C3 hydroxyl groups of the mannose moiety esterified to short- and medium-chain fatty acids (typically C2–C16) [27]. Based on the acetylation degree at these positions and their differential mobility in thin-layer chromatography, MELs are classified into four structural variants: MEL-A (diacetylated, >70% of total MELs), MEL-B (monoacetylated at C6), MEL-C (monoacetylated at C4), and MEL-D (non-acetylated) [28]. A novel tri-acylated variant termed MEL-E was identified in *S. reilianum*, in which an additional short-chain fatty acid (C6–C8) replaces the acetyl groups at C4 or C6 of the mannose moiety, resulting in altered physicochemical properties and highlighting the structural diversity of MELs [29].

**Figure 4:**
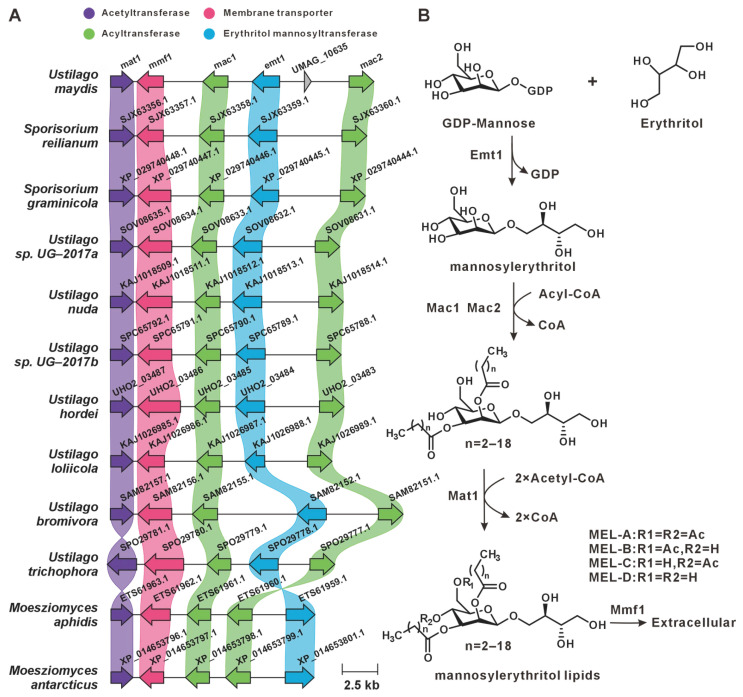
MEL biosynthesis. (**A**) Comparison of the BGC for MELs and its similar BGCs: homologous genes are connected by a band of the same color. (**B**) The biosynthetic pathway for MELs.

Previous studies have characterized the MELs BGC in *U. maydis*, which comprises five core genes [30]. In this study, comparative genomic mining across 16 Ustilaginaceae genomes, including *U. maydis*, revealed that this BGC is widely conserved among 12 species (Figure 4A). The biosynthetic pathway of MELs and the functional roles of individual cluster-encoded genes have been characterized [31,32,33]. We infer that these 12 species possess the genetic capacity for MEL production (Figure 4B).

### 3.4. Siderophores and Non-Ribosomal Peptides

Siderophores constitute a class of low-molecular-weight compounds produced by most microorganisms, exhibiting high affinity for ferric iron acquisition and storage [34]. Assessment of siderophore function in *U. maydis* pathogenicity has demonstrated that high-affinity iron uptake systems are indispensable for virulence [35]. Ferrichrome and ferrichrome A are two siderophore derivatives produced by *U. maydis*, each consisting of a cyclic hexapeptide comprising three δ-N-acyl-N-hydroxyornithine residues and three additional amino acids (glycine, serine, and alanine) [36,37]. Structural diversity among siderophores arises from modifications to the ornithine side chains and variations in the three non-ornithine amino acids within the cyclic peptide backbone [38].

**Figure 5:**
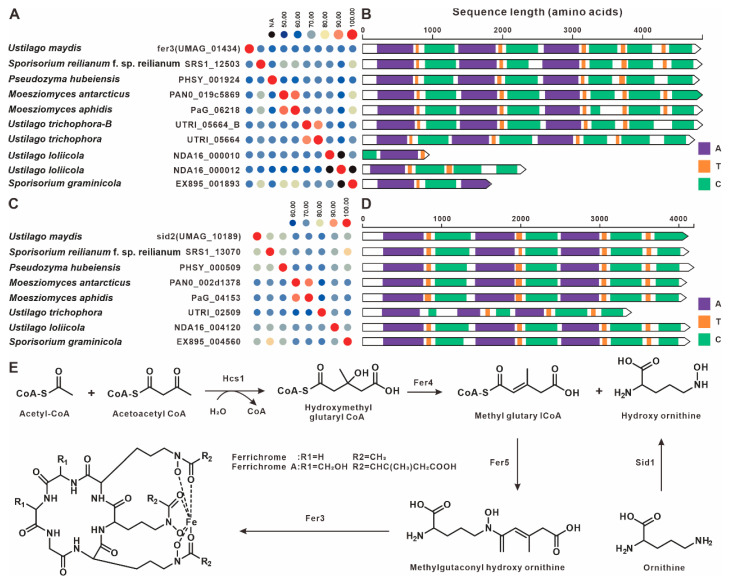
Siderophore compound biosynthesis. (**A**) Comparison of the amino acid sequence identity of fer3 and its homologues. (**B**) Domain comparison of fer3 and its homologues. (**C**) Comparison of the amino acid sequence identity of sid2 and its homologues. The following abbreviations for protein domains are predicted by Synthaser: A, acyltransferase; T, thiolation domain; C, condensation domain. (**D**) Domain comparison of sid2 and its homologues. (**E**) The biosynthetic pathway for Siderophores.

**Figure 6:**
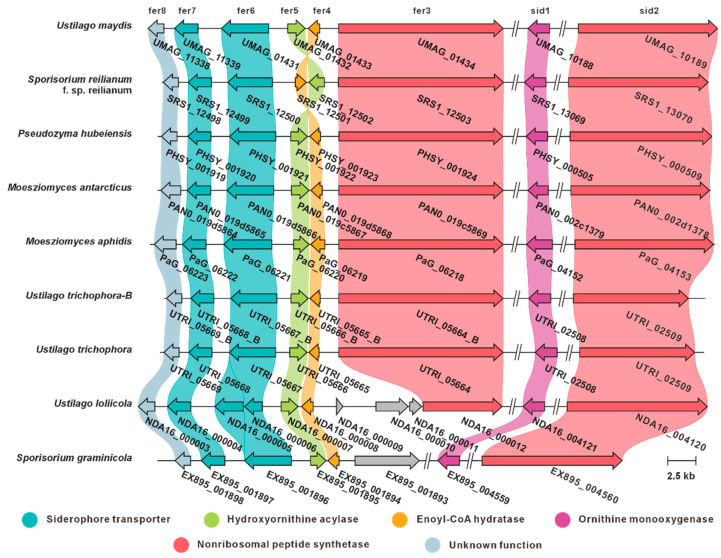
Comparison of the BGC for Siderophores and its similar BGCs. The fer3 and the sid2 are located on different chromosomes in each species.

The siderophore BGC in *U. maydis* comprises six core genes encoding: the NRPS Fer3, enoyl-CoA hydratase Fer4, hydroxyornithine acyltransferase Fer5, two transmembrane transporters (Fer6 and Fer7), and a hypothetical protein Fer8 [39]. In addition, *hcs*1 in *U. maydis* catalyzes the formation of hydroxymethyl glutaryl-CoA from acetyl-CoA and acetoacetyl-CoA [39]. In this study, comparative genomic analysis identified eight *fer*3 paralogs and seven sid2 paralogs across the analyzed genomes. All *fer*3 gene products exhibited >59% pairwise sequence identity (Figure 5A, Table S3), whereas all sid2 homologs demonstrated >63% identity (Figure 5C, Table S4). In *U. maydis*, *sid*2 encodes a multidomain NRPS that acts in concert with the Sid1 L-ornithine-N5-oxygenase to convert precursor amino acids, such as L-ornithine, into the siderophore ferrichrome. Domain architecture analysis revealed highly conserved domain compositions among these paralogs across species (Figure 5B,D). Comparative BGC analysis further indicated that the siderophore BGCs from eight species, including *U. maydis*, share high synteny and sequence similarity (Figure 6). These results suggest that these species possess the genetic capacity for siderophore biosynthesis. The proposed biosynthetic pathway is illustrated in Figure 5E.

### 3.5. Itaconic Acid and Its Biosynthetic Pathway

**Figure 7:**
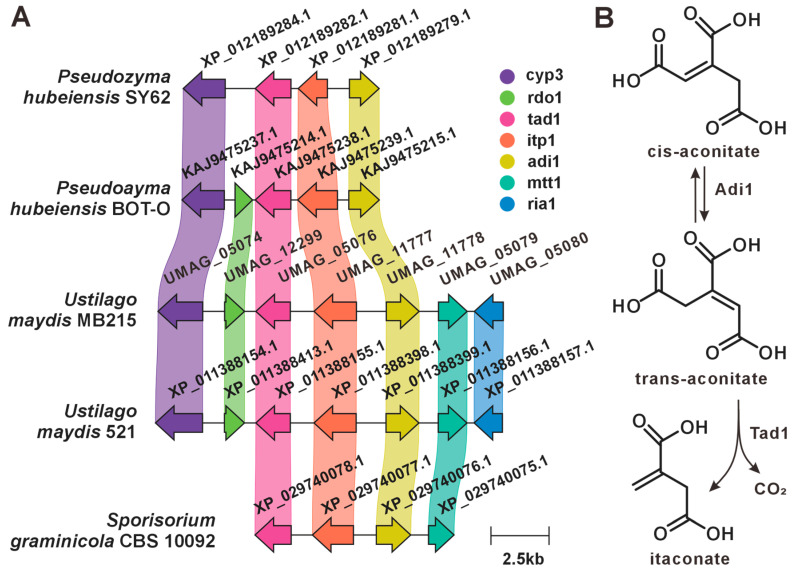
Itaconic acid biosynthesis. (**A**) Comparison of the BGC for itaconic acid and its similar BGCs: homologous genes are connected by a band of the same color. (**B**) The biosynthetic pathway for itaconic acid.

Itaconic acid is an industrially relevant organic compound with diverse biotechnological applications. Historically, itaconic acid was obtained by pyrolytic distillation of citric acid, but current industrial production relies on fermentation by *Aspergillus terreus* [40]. Genomic analysis indicates that *U. maydis* lacks homologs of the A. terreus cis-aconitate decarboxylase CadA (a PrpD family protein), suggesting that *U. maydis* employs an alternative biosynthetic route for itaconate production [41]. Previous studies identified a seven-gene cluster responsible for itaconic acid biosynthesis in the *U. maydis* strain MB215. The gene product UMAG_05079 (Mtt1) functions as a mitochondrial transporter, actively exporting cis-aconitate from mitochondria to the cytoplasm [42]. Subsequently, cis-aconitate is converted to trans-aconitate by the major facilitator superfamily transporter UMAG_11778 (Adi1), followed by decarboxylation to itaconic acid catalyzed by UMAG_05076 (Tad1). Finally, itaconic acid is secreted across the plasma membrane via the transporter UMAG_11777 (Itp1). This BGC additionally encodes UMAG_05074 (Cyp3), which likely catalyzes itaconic acid modification [product unspecified], the functionally uncharacterized protein UMAG_12299 (Rdo1), and the putative transcriptional regulator UMAG_05080 (Ria1) [43]. In this study, we identified highly similar itaconic acid BGCs in four additional species, inferring their genetic capacity for itaconic acid biosynthesis (Figure 7).

### 3.6. Melanin and PKS

**Figure 8:**
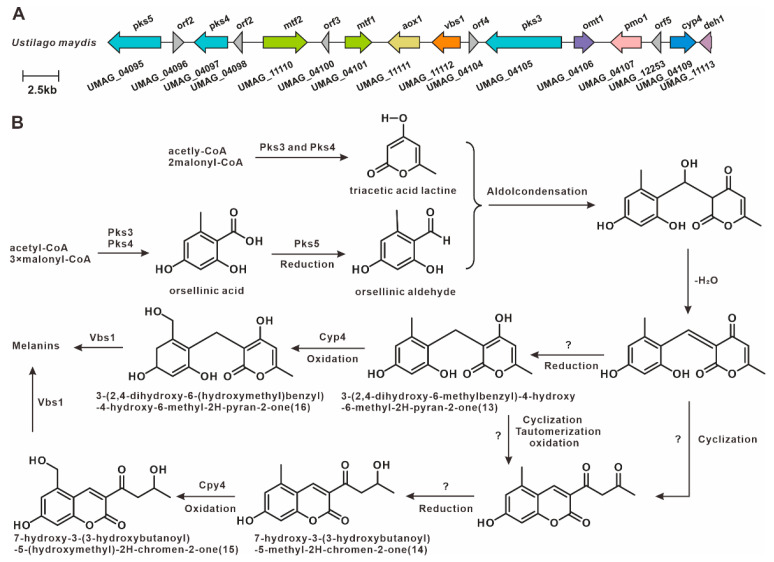
Melanin-associated biosynthesis in *U. maydis* strain 521 [45]. (**A**) Strain-specific PKS gene cluster. (**B**) Proposed melanin biosynthetic pathway.

Previous studies have demonstrated that spore formation in plant tumor-like tissues is associated with melanin biosynthesis [44]. In *U. maydis*, a melanin-associated PKS pathway has been identified. These PKS enzymes synthesize orsellinic acid (OA), triacetic acid lactone (TAL), and related derivatives, which subsequently undergo cyclization and modification to form coumarins and pyran-2-ones [45], ultimately forming this polymer (Figure 8). This study showed that the homologs of the key melanin synthesis genes pks3, pks4, and pks5 were not found in 15 other genomes outside of *U. maydis*, which suggests that this PKS gene cluster is specific to strain 521 and may be the result of horizontal gene transfer.

**Figure 9:**
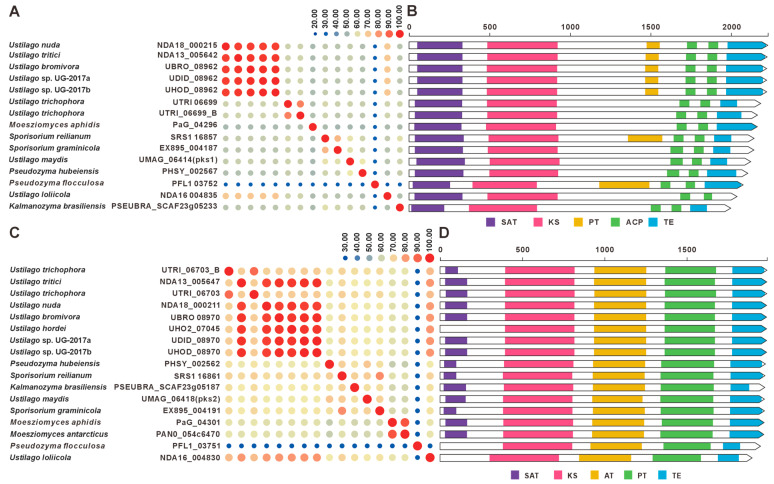
Comparative analysis of pks1 and pks2 with their homologues. (**A**) Comparison of the amino acid sequence identity of pks1 and its homologues. (**B**) Domain comparison of pks1 and its homologues. (**C**) Comparison of the amino acid sequence identity of pks2 and its homologues. (**D**) Domain comparison of pks2 and its homologues. The following abbreviations for protein domains are predicted by synthaser: SAT, starter unit acyltransferase; KS, ketosynthase; AT, acyltransferase; PT, pyranone formation (in PKS); ACP, acyl carrier protein; TE, thioesterase.

**Figure 10:**
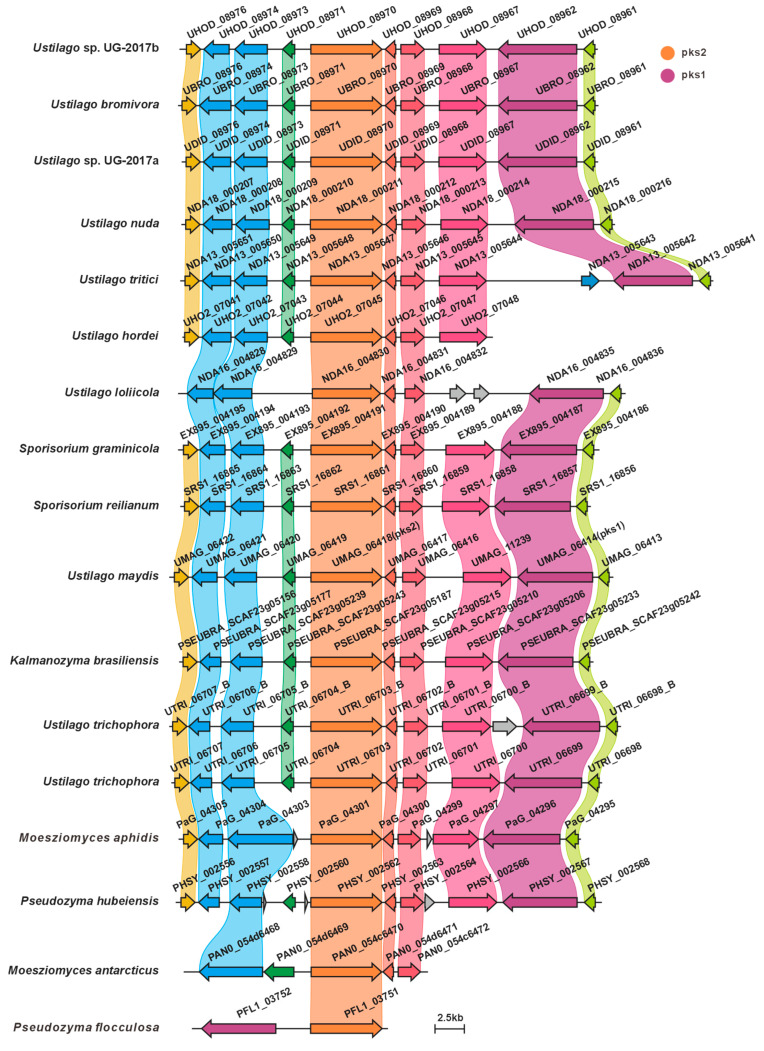
Comparison of putative melanin BGCs from 16 Ustilaginaceae species.

UMAG_06414 (*pks*1) and UMAG_06418 (*pks*2) are polyketide synthase genes involved in melanin biosynthesis in *U. maydis*, and they are located adjacent to each other in the genome. Deletion of *pks*1 alone is sufficient to completely block melanin biosynthesis, indicating that UMAG_06418 cannot compensate for its function, suggesting that the two may together constitute a functionally complete polyketide synthase system. In this study, we identified homologous gene clusters in 15 species other than *U. maydis*. Among them, 13 species harbor homologs of *pks*1, and all 15 species harbor homologs of *pks*2. Sequence similarity comparisons revealed that, with the exception of *P. flocculosa*, the *pks*1 homologs exhibited high sequence similarity (>50%, Figure 9A, Table S5). Similarly, the *pks*2 homologs, also with the exception of *P. flocculosa*, showed high sequence similarity (>58.4%, Figure 9C, Table S6). Domain architecture analysis revealed that the domain organizations of these PKSs are highly conserved (Figure 9B,D). Comparative analysis of the BGCs indicated that most of the 16 species display high synteny and sequence similarity. Among them, *P. flocculosa* showed the lowest similarity compared to the others. In *U. hordei* and *M. antarcticus*, only *pks*2 homologs were present in the gene clusters, suggesting the presence of a putative melanin BGC in these species (Figure 10).

## 4. Discussion

This study systematically analyzed the genomes of 16 species within the family Ustilaginaceae, with particular emphasis on their BGCs. Using antiSMASH 7.0, a total of 181 putative BGCs were identified, averaging approximately 11 BGCs per genome, thereby providing a comprehensive resource for investigating the biosynthetic potential of Ustilaginaceae. BiG-SCAPE network analysis classified these BGCs into 15 gene cluster families (GCFs), revealing distinct distribution patterns of NRPS, PKS, and terpene synthase clusters across the analyzed genomes. Notably, the majority of GCFs comprised BGCs of a single chemical class, suggesting that distinct BGC types may have evolved along relatively independent trajectories. Additionally, network analysis identified potential functional associations among BGCs, offering a framework for subsequent functional characterization and metabolic pathway reconstruction.

The distribution of ustilagic acid BGCs across Ustilaginaceae species was comprehensively analyzed in this study. We identified four species with conserved ustilagic acid BGCs, expanding our understanding of the biosynthetic diversity of this characteristic glycolipid. Previous work had only characterized ustilagic acid production in *U. maydis* and flocculosin in *P. flocculosa* [23,24]; our comparative analysis reveals that this BGC is also conserved in *S. graminicola* and *P. hubeiensis*, suggesting a broader ecological role among related species. MELs, well-characterized biosurfactants produced by Ustilaginaceae, are known to suppress plant immunity in *U. maydis* infection; their BGCs were widely distributed across 12 species, suggesting a conserved role in host manipulation. This high level of conservation aligns with the essential function of MELs in pathogenic development reported for *U. maydis* [31], and indicates that most Ustilaginaceae species likely retain this virulence trait. Siderophores, critical for microbial iron acquisition and virulence, were found to possess highly conserved BGCs across eight species, indicating their essential role in competing for iron in the plant apoplast during infection. This finding underscores the evolutionary conservation of siderophore biosynthesis and its contribution to pathogenicity, consistent with earlier studies [35]. Itaconic acid, an industrially relevant organic compound, has been extensively studied in *U. maydis*, where it contributes to virulence by inhibiting host aconitase. We observed that the itaconic acid BGC in *U. maydis* MB215 exhibits high synteny with BGCs from four additional species, suggesting conserved pathogenic roles and their potential utility as novel production strains. This finding expands upon the known itaconic acid pathway [41,43] and indicates that it is not unique to *U. maydis* but is shared by several other Ustilaginaceae. Melanin-associated PKS pathways, implicated in spore formation within plant tumor-like tissues, have been characterized in *U. maydis* 521. Melanin enhances fungal resistance to plant defense responses and oxidative stress. The strain-specific distribution of these PKS BGCs offers insights into the evolutionary diversification of melanin biosynthesis mechanisms. Additionally, this study identified homologs of the melanin biosynthesis genes *pks*1 and *pks*2 in the remaining 15 Ustilaginaceae species. Both genes are involved in stress tolerance and virulence in *U. maydis* [45]. Most of these homologs exhibited high sequence similarity, with *P. flocculosa* showing the lowest similarity compared to the others. The widespread presence of *pks*1/*pks*2 homologs suggests that the core melanin pathway is ancestral in Ustilaginaceae, in contrast to the strain-specific PKS cluster (*pks*3–*pks*5) found only in *U. maydis* 521. In *U. hordei* and *M. antarcticus*, only *pks*2 homologs were present in the gene clusters, suggesting that these clusters may have been assembled at the edges of contigs during genome assembly.

Collectively, these results directly address our primary objective: to use *U. maydis* as a reference for mapping the distribution of pathogenicity-associated BGCs across Ustilaginaceae. We show that BGCs for MELs, siderophores, itaconic acid, and the core melanin pathway (*pks*1/*pks*2) are widely conserved among most species, whereas a distinct melanin BGC is unique to *U. maydis* 521. These findings are consistent with previous functional studies in *U. maydis* [23,31,35,41,45] and extend them by revealing the phylogenetic distribution of these pathways across the family.

Despite these advances, several limitations should be acknowledged. First, the species selection was constrained by the availability of public genome data at the time of analysis; as more genomes of phytopathogenic Ustilaginaceae become available, future studies should incorporate them to further validate the pathogenicity-associated BGCs identified here. Second, our predictions are purely computational; experimental validation through gene knockouts or heterologous expression is required to confirm the functional roles of the identified BGCs. Third, the presence of a conserved but uncharacterized PKS cluster in 15 species highlights a gap in our understanding of its biological function.

Future research should prioritize the experimental characterization of this uncharacterized PKS cluster using CRISPR-Cas9-mediated genome editing and heterologous expression systems. Such approaches will enable mechanistic dissection of BGC-encoded metabolic pathways and their regulatory networks, ultimately linking genotype to phenotype in plant infection.

In summary, this study provides the first systematic comparative genomic analysis of BGCs across 16 Ustilaginaceae species. Our findings reveal both conserved and species-specific biosynthetic potentials, advancing our understanding of the evolutionary and ecological adaptations within this family. Furthermore, this work establishes a foundation for the discovery and engineering of novel bioactive natural products, as well as for the development of targeted antimicrobial strategies.

## 5. Conclusions

The dissemination of plant-pathogenic fungi poses a severe threat to global agricultural production, with their virulence intimately linked to secondary metabolite biosynthesis. This study employed comparative genomic and bioinformatic approaches to systematically characterize the distribution and architecture of BGCs across 16 species within the family Ustilaginaceae. Our findings reveal that BGCs governing the production of ustilagic acid, MELs, and siderophores are widely conserved among Ustilaginaceae. Notably, a strain-specific PKS gene cluster associated with melanin biosynthesis was exclusively identified in the *U. maydis* strain 521. Meanwhile, putative melanin biosynthetic gene clusters were identified in the remaining 15 species of the Ustilaginaceae. This research provides a framework for elucidating pathogenic mechanisms and metabolic regulation in Ustilaginaceae, establishing a theoretical foundation for future synthetic biology applications in antimicrobial development and high-value natural product engineering.

## Data Availability

All summary data were provided in the results and Supplemental Files.

## Supplementary Materials

The following supporting information can be downloaded at: https://www.mdpi.com/article/10.3390/jof12050319/s1, Figure S1: GCF network of Ustilaginaceae BGCs. Triangles and circles represent reference BGCs from the MIBiG database and BGCs identified from the 16 analyzed Ustilaginaceae genomes, respectively. Distinct colors indicate different BGC classes: green for NRPS, purple for terpene synthases, and orange for PKS; Table S1: Ustilaginaceae species and genome information used in this study; Table S2: List of predicted biosynthetic gene clusters (BGCs) in Ustilaginaceae strains; Table S3: Sequence identity matrix of fas2 and its orthologs; Table S4: Sequence identity matrix of fer3 and its orthologs; Table S5: Sequence identity matrix of sid2 and its orthologs; Table S6: Sequence identity matrix of pks1 and its orthologs; Table S7: Sequence identity matrix of pks2 and its orthologs.
