# Supplementary material for: Biosynthetic Gene Cluster Diversity and Species-Specific Metabolic Potential in Ustilaginaceae
Source: J Fungi (Basel). 2026 Apr 27;12(5):319. doi: 10.3390/jof12050319 (PMC13208730; doi:10.3390/jof12050319)
Supplement: Supplementary file 1 [file jof-12-00319-s001.zip › jof-4216461-supplementary.pdf]

Supplementary Figure

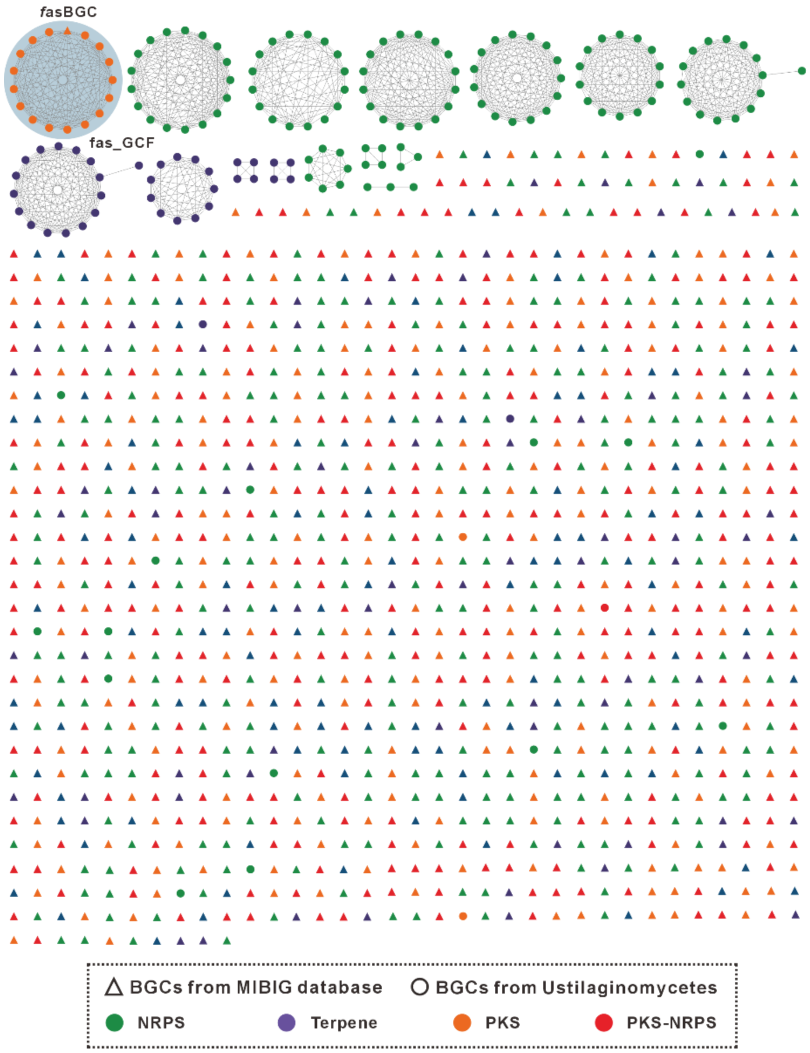

Figure S1. GCF network of Ustilaginaceae BGCs. Triangles and circles represent reference BGCs from the MIBiG database and BGCs identified from the 16 analyzed Ustilaginaceae genomes, respectively. Distinct colors indicate different BGC classes: green for NRPS, purple for terpene synthases, and orange for PKS.

Supplementary Table

Table S1. Ustilaginaceae species and genome information used in this study.

Species	Accession No.
Ustilago maydis 521	GCF_000328475.2
Ustilago trichophora	GCA_900323505.1
Ustilago nuda	GCA_022963125.1
Ustilago loliicola	GCA_022963135.1
Ustilago hordei	GCF_900519145.1
Ustilago bromivora	GCA_900080155.1
Ustilago sp. UG-2017a	GCA_900242705.1
Ustilago sp. UG-2017b	GCA_900242695.1
Sporisorium reilianum	GCA_900162835.1
Kalmanozyma brasiliensis	GCF_000497045.1
Moesziomyces antarcticus	GCF_000747765.1
Moesziomyces aphidis	GCA_000517465.1
Pseudozyma flocculosa	GCF_000417875.1
Pseudozyma hubeiensis	GCF_000403515.1
Sporisorium graminicola	GCF_005498985.1
Ustilago tritici	GCA_022963115.1

Table S2. List of predicted biosynthetic gene clusters (BGCs) in Ustilaginaceae strains.

BGC	Accession ID	Product Prediction	BiG-SCAPE class	Organism
AWNI01000002.1.region001	AWNI01000002.1	NRPS-like	NRPS	Moesziomyces aphidis
AWNI01000004.1.region001	AWNI01000004.1	NRPS-like	NRPS	Moesziomyces aphidis
AWNI01000008.1.region001	AWNI01000008.1	NRPS-like	NRPS	Moesziomyces aphidis
AWNI01000009.1.region001	AWNI01000009.1	NRPS-like	NRPS	Moesziomyces aphidis
AWNI01000009.1.region002	AWNI01000009.1	NRPS-like	NRPS	Moesziomyces aphidis
AWNI01000011.1.region001	AWNI01000011.1	terpene	Terpene	Moesziomyces aphidis
AWNI01000012.1.region001	AWNI01000012.1	NRPS-like	NRPS	Moesziomyces aphidis
AWNI01000014.1.region001	AWNI01000014.1	NRPS	NRPS	Moesziomyces aphidis
AWNI01000015.1.region001	AWNI01000015.1	T1PKS	PKSI	Moesziomyces aphidis
AWNI01000022.1.region001	AWNI01000022.1	terpene	Terpene	Moesziomyces aphidis
AWNI01000040.1.region001	AWNI01000040.1	terpene	Terpene	Moesziomyces aphidis
AWNI01000041.1.region001	AWNI01000041.1	NRPS	NRPS	Moesziomyces aphidis
JALCUS010000002.1.region001	JALCUS010000002.1	T1PKS	PKSI	Ustilago nuda
JALCUS010000007.1.region001	JALCUS010000007.1	NRPS-like	NRPS	Ustilago nuda
JALCUS010000014.1.region001	JALCUS010000014.1	NRPS-like	NRPS	Ustilago nuda
JALCUS010000014.1.region002	JALCUS010000014.1	NRPS-like	NRPS	Ustilago nuda
JALCUS010000016.1.region001	JALCUS010000016.1	NRPS	NRPS	Ustilago nuda
JALCUS010000016.1.region002	JALCUS010000016.1	terpene	Terpene	Ustilago nuda
JALCUS010000023.1.region001	JALCUS010000023.1	NRPS	NRPS	Ustilago nuda
JALCUS010000026.1.region001	JALCUS010000026.1	terpene	Terpene	Ustilago nuda
JALCUS010000028.1.region001	JALCUS010000028.1	NRPS-like	NRPS	Ustilago nuda
JALCUT010000012.1.region001	JALCUT010000012.1	NRPS-like	NRPS	Ustilago tritici
JALCUT010000012.1.region002	JALCUT010000012.1	NRPS-like	NRPS	Ustilago tritici
JALCUT010000013.1.region001	JALCUT010000013.1	NRPS-like	NRPS	Ustilago tritici
JALCUT010000014.1.region001	JALCUT010000014.1	NRPS-like	NRPS	Ustilago tritici
JALCUT010000015.1.region001	JALCUT010000015.1	NRPS-like	NRPS	Ustilago tritici
JALCUT010000016.1.region001	JALCUT010000016.1	terpene	Terpene	Ustilago tritici
JALCUT010000016.1.region002	JALCUT010000016.1	NRPS	NRPS	Ustilago tritici
JALCUT010000019.1.region001	JALCUT010000019.1	terpene	Terpene	Ustilago tritici
JALCUT010000022.1.region001	JALCUT010000022.1	T1PKS	PKSI	Ustilago tritici
JALCUT010000025.1.region001	JALCUT010000025.1	NRPS	NRPS	Ustilago tritici
JALCUU010000001.1.region001	JALCUU010000001.1	NRPS.NRPS-like	NRPS	Ustilago loliicola
JALCUU010000012.1.region001	JALCUU010000012.1	NRPS-like	NRPS	Ustilago loliicola
JALCUU010000013.1.region001	JALCUU010000013.1	NRPS-like	NRPS	Ustilago loliicola
JALCUU010000013.1.region002	JALCUU010000013.1	NRPS-like	NRPS	Ustilago loliicola
JALCUU010000017.1.region001	JALCUU010000017.1	NRPS-like	NRPS	Ustilago loliicola
JALCUU010000022.1.region001	JALCUU010000022.1	terpene	Terpene	Ustilago loliicola
JALCUU010000035.1.region001	JALCUU010000035.1	terpene	Terpene	Ustilago loliicola
JALCUU010000035.1.region002	JALCUU010000035.1	NRPS	NRPS	Ustilago loliicola
JALCUU010000035.1.region003	JALCUU010000035.1	NRPS	NRPS	Ustilago loliicola
JALCUU010000039.1.region001	JALCUU010000039.1	T1PKS	PKSI	Ustilago loliicola

LT558117.1.region001	LT558117.1	NRPS-like	NRPS	Ustilago bromivora
LT558120.1.region001	LT558120.1	NRPS-like	NRPS	Ustilago bromivora
LT558120.1.region002	LT558120.1	NRPS-like	NRPS	Ustilago bromivora
LT558121.1.region001	LT558121.1	terpene	Terpene	Ustilago bromivora
LT558121.1.region002	LT558121.1	NRPS	NRPS	Ustilago bromivora
LT558122.1.region001	LT558122.1	NRPS-like	NRPS	Ustilago bromivora
LT558123.1.region001	LT558123.1	NRPS-like	NRPS	Ustilago bromivora
LT558127.1.region001	LT558127.1	NRPS	NRPS	Ustilago bromivora
LT558129.1.region001	LT558129.1	terpene	Terpene	Ustilago bromivora
LT558139.1.region001	LT558139.1	T1PKS	PKSI	Ustilago bromivora
LT795054.1.region001	LT795054.1	NRPS-like	NRPS	Sporisorium reilianum
LT795055.1.region001	LT795055.1	NRPS	NRPS	Sporisorium reilianum
LT795056.1.region001	LT795056.1	NRPS-like	NRPS	Sporisorium reilianum
LT795056.1.region002	LT795056.1	NRPS-like	NRPS	Sporisorium reilianum
LT795057.1.region001	LT795057.1	NRPS	NRPS	Sporisorium reilianum
LT795057.1.region002	LT795057.1	terpene	Terpene	Sporisorium reilianum
LT795059.1.region001	LT795059.1	NRPS-like	NRPS	Sporisorium reilianum
LT795060.1.region001	LT795060.1	NRPS-like	NRPS	Sporisorium reilianum
LT795067.1.region001	LT795067.1	terpene	Terpene	Sporisorium reilianum
LT795070.1.region001	LT795070.1	NRPS-like	NRPS	Sporisorium reilianum
LT795076.1.region001	LT795076.1	T1PKS	PKSI	Sporisorium reilianum
NC_026478.1.region001	NC_026478.1	NRPS-like	NRPS	Ustilago maydis 521
NC_026479.1.region001	NC_026479.1	NRPS	NRPS	Ustilago maydis 521
NC_026480.1.region001	NC_026480.1	NRPS-like	NRPS	Ustilago maydis 521
NC_026480.1.region002	NC_026480.1	NRPS-like	NRPS	Ustilago maydis 521
NC_026481.1.region001	NC_026481.1	NRPS	NRPS	Ustilago maydis 521
NC_026481.1.region002	NC_026481.1	terpene	Terpene	Ustilago maydis 521
NC_026483.1.region001	NC_026483.1	NRPS-like	NRPS	Ustilago maydis 521
NC_026484.1.region001	NC_026484.1	NRPS-like	NRPS	Ustilago maydis 521
NC_026489.1.region001	NC_026489.1	NRPS-like	NRPS	Ustilago maydis 521
NC_026491.1.region001	NC_026491.1	terpene	Terpene	Ustilago maydis 521
NC_026494.1.region001	NC_026494.1	NRPS-like	NRPS	Ustilago maydis 521
NC_026496.1.region001	NC_026496.1	NRPS	NRPS	Ustilago maydis 521
NC_026500.1.region001	NC_026500.1	T1PKS	PKSI	Ustilago maydis 521
NC_043719.1.region001	NC_043719.1	NRPS-like	NRPS	Sporisorium graminicola
NC_043722.1.region001	NC_043722.1	NRPS	NRPS	Sporisorium graminicola
NC_043724.1.region001	NC_043724.1	terpene	Terpene	Sporisorium graminicola
NC_043725.1.region001	NC_043725.1	NRPS-like	NRPS	Sporisorium graminicola
NC_043728.1.region001	NC_043728.1	NRPS	NRPS	Sporisorium graminicola
NC_043729.1.region001	NC_043729.1	terpene	Terpene	Sporisorium graminicola
NC_043731.1.region001	NC_043731.1	NRPS.NRPS-like	NRPS	Sporisorium graminicola
NC_043731.1.region002	NC_043731.1	NRPS-like	NRPS	Sporisorium graminicola
NC_043732.1.region001	NC_043732.1	T1PKS	PKSI	Sporisorium graminicola

NC_043733.1.region001	NC_043733.1	T1PKS	PKSI	Sporisorium graminicola
NC_043734.1.region001	NC_043734.1	NRPS	NRPS	Sporisorium graminicola
NC_043734.1.region002	NC_043734.1	terpene	Terpene	Sporisorium graminicola
NC_043736.1.region001	NC_043736.1	NRPS-like	NRPS	Sporisorium graminicola
NC_043736.1.region002	NC_043736.1	NRPS-like	NRPS	Sporisorium graminicola
NW_006920869.1.region001	NW_006920869.1	NRPS-like.T1PKS	PKS-NRP_Hybrids	Pseudozyma flocculosa PF-1
NW_006920869.1.region002	NW_006920869.1	fungal-RiPP	RiPPs	Pseudozyma flocculosa PF-1
NW_006920871.1.region001	NW_006920871.1	NRPS-like	NRPS	Pseudozyma flocculosa PF-1
NW_006920874.1.region001	NW_006920874.1	NRPS-like	NRPS	Pseudozyma flocculosa PF-1
NW_006920877.1.region001	NW_006920877.1	terpene	Terpene	Pseudozyma flocculosa PF-1
NW_006920877.1.region002	NW_006920877.1	NRPS	NRPS	Pseudozyma flocculosa PF-1
NW_006920878.1.region001	NW_006920878.1	NRPS-like	NRPS	Pseudozyma flocculosa PF-1
NW_006920879.1.region001	NW_006920879.1	NRPS-like	NRPS	Pseudozyma flocculosa PF-1
NW_006920879.1.region002	NW_006920879.1	T1PKS	PKSI	Pseudozyma flocculosa PF-1
NW_006920889.1.region001	NW_006920889.1	terpene	Terpene	Pseudozyma flocculosa PF-1
NW_006920892.1.region001	NW_006920892.1	NRPS-like	NRPS	Pseudozyma flocculosa PF-1
NW_012133790.1.region001	NW_012133790.1	NRPS	NRPS	Pseudozyma hubeiensis SY62
NW_012133795.1.region001	NW_012133795.1	NRPS-like	NRPS	Pseudozyma hubeiensis SY62
NW_012133803.1.region001	NW_012133803.1	NRPS	NRPS	Pseudozyma hubeiensis SY62
NW_012133806.1.region001	NW_012133806.1	terpene	Terpene	Pseudozyma hubeiensis SY62
NW_012133807.1.region001	NW_012133807.1	T1PKS	PKSI	Pseudozyma hubeiensis SY62
NW_012133815.1.region001	NW_012133815.1	NRPS	NRPS	Pseudozyma hubeiensis SY62
NW_012133820.1.region001	NW_012133820.1	NRPS-like	NRPS	Pseudozyma hubeiensis SY62
NW_012133820.1.region002	NW_012133820.1	NRPS-like	NRPS	Pseudozyma hubeiensis SY62
NW_012133821.1.region001	NW_012133821.1	NRPS-like	NRPS	Pseudozyma hubeiensis SY62
NW_012133834.1.region001	NW_012133834.1	terpene	Terpene	Pseudozyma hubeiensis SY62
NW_012133850.1.region001	NW_012133850.1	NRPS-like	NRPS	Pseudozyma hubeiensis SY62
NW_014638930.1.region001	NW_014638930.1	NRPS-like	NRPS	Moesziomyces antarcticus
NW_014638956.1.region001	NW_014638956.1	T1PKS	PKSI	Moesziomyces antarcticus
NW_014638989.1.region001	NW_014638989.1	terpene	Terpene	Moesziomyces antarcticus
NW_014638991.1.region001	NW_014638991.1	NRPS	NRPS	Moesziomyces antarcticus
NW_014638998.1.region001	NW_014638998.1	terpene	Terpene	Moesziomyces antarcticus
NW_014639005.1.region001	NW_014639005.1	NRPS-like	NRPS	Moesziomyces antarcticus
NW_014639006.1.region001	NW_014639006.1	terpene	Terpene	Moesziomyces antarcticus
NW_014639007.1.region001	NW_014639007.1	NRPS-like	NRPS	Moesziomyces antarcticus
NW_014639007.1.region002	NW_014639007.1	NRPS-like	NRPS	Moesziomyces antarcticus
NW_014639008.1.region001	NW_014639008.1	NRPS	NRPS	Moesziomyces antarcticus
NW_014639009.1.region001	NW_014639009.1	NRPS-like	NRPS	Moesziomyces antarcticus
NW_015961950.1.region001	NW_015961950.1	terpene	Terpene	Kalmanozyma brasiliensis GHG001
NW_015961954.1.region001	NW_015961954.1	NRPS-like	NRPS	Kalmanozyma brasiliensis GHG001
NW_015961955.1.region001	NW_015961955.1	NRPS	NRPS	Kalmanozyma brasiliensis GHG001
NW_015961959.1.region001	NW_015961959.1	NRPS-like	NRPS	Kalmanozyma brasiliensis GHG001
NW_015961959.1.region002	NW_015961959.1	NRPS-like	NRPS	Kalmanozyma brasiliensis GHG001

NW_015961962.1.region001	NW_015961962.1	terpene	Terpene	Kalmanozyma brasiliensis GHG001
NW_015961963.1.region001	NW_015961963.1	T1PKS	PKSI	Kalmanozyma brasiliensis GHG001
NW_015961970.1.region001	NW_015961970.1	NRPS-like	NRPS	Kalmanozyma brasiliensis GHG001
NW_015961988.1.region001	NW_015961988.1	NRPS-like	NRPS	Kalmanozyma brasiliensis GHG001
NW_015961989.1.region001	NW_015961989.1	NRPS-like	NRPS	Kalmanozyma brasiliensis GHG001
NW_024545253.1.region001	NW_024545253.1	NRPS-like	NRPS	Ustilago hordei
NW_024545261.1.region001	NW_024545261.1	NRPS-like	NRPS	Ustilago hordei
NW_024545261.1.region002	NW_024545261.1	NRPS-like	NRPS	Ustilago hordei
NW_024545265.1.region001	NW_024545265.1	T1PKS	PKSI	Ustilago hordei
NW_024545270.1.region001	NW_024545270.1	NRPS-like	NRPS	Ustilago hordei
NW_024545279.1.region001	NW_024545279.1	NRPS-like	NRPS	Ustilago hordei
NW_024545280.1.region001	NW_024545280.1	terpene	Terpene	Ustilago hordei
NW_024545283.1.region001	NW_024545283.1	terpene	Terpene	Ustilago hordei
NW_024545290.1.region001	NW_024545290.1	NRPS	NRPS	Ustilago hordei
NW_024545292.1.region001	NW_024545292.1	NRPS	NRPS	Ustilago hordei
OEYX01000001.1.region001	OEYX01000001.1	NRPS-like	NRPS	Ustilago sp. UG-2017a
OEYX01000002.1.region001	OEYX01000002.1	terpene	Terpene	Ustilago sp. UG-2017a
OEYX01000031.1.region001	OEYX01000031.1	NRPS-like	NRPS	Ustilago sp. UG-2017a
OEYX01000057.1.region001	OEYX01000057.1	NRPS-like	NRPS	Ustilago sp. UG-2017a
OEYX01000057.1.region002	OEYX01000057.1	NRPS-like	NRPS	Ustilago sp. UG-2017a
OEYX01000058.1.region001	OEYX01000058.1	NRPS	NRPS	Ustilago sp. UG-2017a
OEYX01000058.1.region002	OEYX01000058.1	terpene	Terpene	Ustilago sp. UG-2017a
OEYX01000116.1.region001	OEYX01000116.1	NRPS-like	NRPS	Ustilago sp. UG-2017a
OEYX01000118.1.region001	OEYX01000118.1	NRPS-like	NRPS	Ustilago sp. UG-2017a
OEYX01000121.1.region001	OEYX01000121.1	T1PKS	PKSI	Ustilago sp. UG-2017a
OHJM01000002.1.region001	OHJM01000002.1	NRPS	NRPS	Ustilago sp. UG-2017b
OHJM01000002.1.region002	OHJM01000002.1	NRPS	NRPS	Ustilago sp. UG-2017b
OHJM01000002.1.region003	OHJM01000002.1	terpene	Terpene	Ustilago sp. UG-2017b
OHJM01000009.1.region001	OHJM01000009.1	NRPS-like	NRPS	Ustilago sp. UG-2017b
OHJM01000018.1.region001	OHJM01000018.1	NRPS-like	NRPS	Ustilago sp. UG-2017b
OHJM01000028.1.region001	OHJM01000028.1	NRPS-like	NRPS	Ustilago sp. UG-2017b
OHJM01000037.1.region001	OHJM01000037.1	NRPS-like	NRPS	Ustilago sp. UG-2017b
OHJM01000037.1.region002	OHJM01000037.1	NRPS-like	NRPS	Ustilago sp. UG-2017b
OHJM01000039.1.region001	OHJM01000039.1	terpene	Terpene	Ustilago sp. UG-2017b
OHJM01000041.1.region001	OHJM01000041.1	T1PKS	PKSI	Ustilago sp. UG-2017b
OOIN01000001.1.region001	OOIN01000001.1	NRPS-like	NRPS	Ustilago trichophora
OOIN01000002.1.region001	OOIN01000002.1	NRPS-like	NRPS	Ustilago trichophora
OOIN01000003.1.region001	OOIN01000003.1	NRPS-like	NRPS	Ustilago trichophora
OOIN01000003.1.region002	OOIN01000003.1	NRPS-like	NRPS	Ustilago trichophora
OOIN01000005.1.region001	OOIN01000005.1	NRPS-like	NRPS	Ustilago trichophora
OOIN01000005.1.region002	OOIN01000005.1	NRPS-like	NRPS	Ustilago trichophora
OOIN01000006.1.region001	OOIN01000006.1	NRPS-like	NRPS	Ustilago trichophora
OOIN01000007.1.region001	OOIN01000007.1	NRPS-like	NRPS	Ustilago trichophora

OOIN01000010.1.region001	OOIN01000010.1	terpene	Terpene	Ustilago trichophora
OOIN01000011.1.region001	OOIN01000011.1	NRPS-like	NRPS	Ustilago trichophora
OOIN01000012.1.region001	OOIN01000012.1	NRPS-like	NRPS	Ustilago trichophora
OOIN01000013.1.region001	OOIN01000013.1	terpene	Terpene	Ustilago trichophora
OOIN01000013.1.region002	OOIN01000013.1	NRPS	NRPS	Ustilago trichophora
OOIN01000018.1.region001	OOIN01000018.1	NRPS	NRPS	Ustilago trichophora
OOIN01000029.1.region001	OOIN01000029.1	terpene	Terpene	Ustilago trichophora
OOIN01000032.1.region001	OOIN01000032.1	NRPS	NRPS	Ustilago trichophora
OOIN01000035.1.region001	OOIN01000035.1	terpene	Terpene	Ustilago trichophora
OOIN01000041.1.region001	OOIN01000041.1	T1PKS	PKSI	Ustilago trichophora
OOIN01000042.1.region001	OOIN01000042.1	T1PKS	PKSI	Ustilago trichophora

Table S3. Sequence identity matrix of fas2 and its orthologs.

fas2	PHSY_000312	PFL1_00007	EX895_002632	UHO2_03648	NDA18_006500	PAN0_010d3997	UHOD_04672	UDID_04672	SRS1_14030	UTRI_03768	NDA16_002164	PaG_03660
100	80.1	53.77	73.1	45.07	44.89	45.72	45.19	45.28	45.45	45.6	45.38	45.76
80.1	100	53.99	72.48	44.69	44.58	45.34	44.78	44.84	45.01	45.03	45.21	45.38
53.77	53.99	100	54.54	50.19	50.18	50.64	50.33	50.38	50.63	50.4	50.66	50.71
73.1	72.48	54.54	100	46.08	46	46.82	46.29	46.29	46.41	46.65	46.66	46.8
45.07	44.69	50.19	46.08	100	99.33	93.3	99.13	99.13	93.81	94.86	96.61	93.28
44.89	44.58	50.18	46	99.33	100	92.93	98.66	98.66	93.44	94.57	96.22	92.91
45.72	45.34	50.64	46.82	93.3	92.93	100	93.2	93.25	94.42	94.62	93.78	99.41
45.19	44.78	50.33	46.29	99.13	98.66	93.2	100	99.64	93.71	95.01	96.61	93.17
45.28	44.84	50.38	46.29	99.13	98.66	93.25	99.64	100	93.76	95.01	96.66	93.23
45.45	45.01	50.63	46.41	93.81	93.44	94.42	93.71	93.76	100	95.32	94.23	94.32
45.6	45.03	50.4	46.65	94.86	94.57	94.62	95.01	95.01	95.32	100	95.57	94.57
45.38	45.21	50.66	46.66	96.61	96.22	93.78	96.61	96.66	94.23	95.57	100	93.72
45.76	45.38	50.71	46.8	93.28	92.91	99.41	93.17	93.23	94.32	94.57	93.72	100

Table S4. Sequence identity matrix of fer3 and its orthologs.

fer3	SRS1_12503	PHSY_001924	PAN0_019c5869	PaG_06218	UTRI_05664_B	UTRI_05664	NDA16_000010	NDA16_000012	EX895_001893
100	64.55	61.51	63.14	62.83	63.7	63.68	64.38	63.17	63.73
64.55	100	63.03	73.26	73	66.31	66.62	65.52	61.86	76.35
61.51	63.03	100	61.15	61.09	64.58	64.77	63.89	59.26	62.64
63.14	73.26	61.15	100	93.08	64.41	64.74	65.53	60.17	77.12
62.83	73	61.09	93.08	100	64.26	64.78	65.1	59.56	77.28
63.7	66.31	64.58	64.41	64.26	100	90.19	64.09	61.05	66.23
63.68	66.62	64.77	64.74	64.78	90.19	100	64.79	60.96	67.05
64.38	65.52	63.89	65.53	65.1	64.09	64.79	100	NA	67.42
63.17	61.86	59.26	60.17	59.56	61.05	60.96	NA	100	NA
63.73	76.35	62.64	77.12	77.28	66.23	67.05	67.42	NA	100

Table S5. Sequence identity matrix of sid2 and its orthologs.

sid2	SRS1_13070	PHSY_000509	PAN0_002d1378	PaG_04153	UTRI_02509	NDA16_004120	EX895_004560
100	77.3	76.94	68.12	67.97	66.95	72.56	75.5
77.3	100	76.64	69.75	69.69	68.87	74.9	84.06
76.94	76.64	100	67.86	67.7	67.52	73.03	75.77
68.12	69.75	67.86	100	92.81	63.61	70.5	68.61
67.97	69.69	67.7	92.81	100	63.37	70.52	68.64
66.95	68.87	67.52	63.61	63.37	100	71.38	67.49
72.56	74.9	73.03	70.5	70.52	71.38	100	73.48
75.5	84.06	75.77	68.61	68.64	67.49	73.48	100

Table S6. Sequence identity matrix of pks1 and its orthologs.

NDA18_00215	NDA13_05642	UBRO_08962	UDID_08962	UHOD_08962	UTRI_06699	UTRI_06699_B	PaG_04296	SRS1_16857	EX895_04187	UMAG_06414	PHSY_02567	PFL1_03752	NDA16_04835	PSEUBRA_SCAF23g05233
100	96.35	96.57	96.44	96.44	56.8	56.97	50.77	54.94	53.67	55.57	52.36	28.9	69.63	53.62
96.35	100	97.75	97.52	97.43	57.18	57.55	51.43	55.36	54	56.05	52.7	29.34	70.38	54.07
96.57	97.75	100	98.78	98.69	57.22	57.58	51.46	55.53	53.98	56.13	52.77	29.23	70.04	54.1
96.44	97.52	98.78	100	99.46	57.03	57.34	51.6	55.34	53.69	55.74	52.63	29.08	70.29	54
96.44	97.43	98.69	99.46	100	57.08	57.44	51.5	55.44	53.74	55.74	52.68	29.23	70.39	54.05
56.8	57.18	57.22	57.03	57.08	100	84.02	52.68	58.27	56.4	56.85	55.76	28.47	57.3	57.59
56.97	57.55	57.58	57.34	57.44	84.02	100	52.52	57.09	54.51	56	54.52	28.25	57.56	56.19
50.77	51.43	51.46	51.6	51.5	52.68	52.52	100	51.33	50	50.36	49.68	29.26	51.34	51.17
54.94	55.36	55.53	55.34	55.44	58.27	57.09	51.33	100	73.44	61.27	57.64	29.47	55.25	59.9
53.67	54	53.98	53.69	53.74	56.4	54.51	50	73.44	100	59.9	56.04	28.39	53.5	57.81
55.57	56.05	56.13	55.74	55.74	56.85	56	50.36	61.27	59.9	100	62.34	29.53	53.9	57.22
52.36	52.7	52.77	52.63	52.68	55.76	54.52	49.68	57.64	56.04	62.34	100	29.2	52.33	55.74
28.9	29.34	29.23	29.08	29.23	28.47	28.25	29.26	29.47	28.39	29.53	29.2	100	26.64	29.51
69.63	70.38	70.04	70.29	70.39	57.3	57.56	51.34	55.25	53.5	53.9	52.33	26.64	100	52.56
53.62	54.07	54.1	54	54.05	57.59	56.19	51.17	59.9	57.81	57.22	55.74	29.51	52.56	100

Table S7. Sequence identity matrix of pks2 and its orthologs.

UTRI_06703_B	NDA13_005647	UTRI_06703	NDA18_000211	UBRO_08970	UHO2_07045	UDID_08970	UHOD_08970	PHSY_002562	SRS1_16861	PSEUBRA_SCAF23g05187	UMAG_06418	EX895_004191	PaG_04301	PAN0_054c6470	PFL1_03751	NDA16_004830
100	70.8	88.92	70.33	70.84	70.84	71.04	71.19	67.14	69.83	68.05	67.84	68.66	63.7	63.87	31.85	74.4
70.8	100	71.15	95.92	98.24	97.38	98.54	98.39	64.53	68.84	66	65.02	66.29	62.69	62.96	31.42	81.03
88.92	71.15	100	70.58	71.09	71.09	71.34	71.39	66.97	70.89	68.56	67.93	69.21	64.01	64.02	32.01	74.03
70.33	95.92	70.58	100	96.12	97.78	96.27	96.27	64.3	68.57	65.88	65.1	66.07	62.67	62.99	31.66	80.01
70.84	98.24	71.09	96.12	100	97.73	98.89	98.94	64.61	68.98	66.24	65.1	66.58	62.88	63.2	31.28	81.07
70.84	97.38	71.09	97.78	97.73	100	97.83	97.88	64.56	68.93	65.88	65.1	66.43	62.83	63.14	31.55	80.91
71.04	98.54	71.34	96.27	98.89	97.83	100	99.65	64.71	69.03	66.19	65.36	66.48	62.98	63.25	31.55	81.34
71.19	98.39	71.39	96.27	98.94	97.88	99.65	100	64.71	69.03	66.14	65.31	66.63	63.13	63.4	31.49	81.44
67.14	64.53	66.97	64.3	64.61	64.56	64.71	64.71	100	70.25	67.24	72.26	67.7	58.4	58.59	31.65	65.35
69.83	68.84	70.89	68.57	68.98	68.93	69.03	69.03	70.25	100	70.39	71.17	82.82	62.91	62.99	32.9	69.8
68.05	66	68.56	65.88	66.24	65.88	66.19	66.14	67.24	70.39	100	67.72	68.15	60.64	60.68	32.08	67.86
67.84	65.02	67.93	65.1	65.1	65.1	65.36	65.31	72.26	71.17	67.72	100	70.08	60.73	60.76	32.04	66.4
68.66	66.29	69.21	66.07	66.58	66.43	66.48	66.63	67.7	82.82	68.15	70.08	100	61.38	61.41	32.02	67.36
63.7	62.69	64.01	62.67	62.88	62.83	62.98	63.13	58.4	62.91	60.64	60.73	61.38	100	92.98	31.45	64.12
63.87	62.96	64.02	62.99	63.2	63.14	63.25	63.4	58.59	62.99	60.68	60.76	61.41	92.98	100	31.81	64.15
31.85	31.42	32.01	31.66	31.28	31.55	31.55	31.49	31.65	32.9	32.08	32.04	32.02	31.45	31.81	100	32.62
74.4	81.03	74.03	80.01	81.07	80.91	81.34	81.44	65.35	69.8	67.86	66.4	67.36	64.12	64.15	32.62	100
